# Carbohydrate Antigen 125 (CA 125): A Novel Biomarker in Acute Heart Failure

**DOI:** 10.3390/diagnostics14080795

**Published:** 2024-04-10

**Authors:** Mihai Cristian Marinescu, Violeta Diana Oprea, Sorina Nicoleta Munteanu, Aurel Nechita, Dana Tutunaru, Luiza Camelia Nechita, Aurelia Romila

**Affiliations:** 1 Faculty of Medicine and Pharmacy, “Dunărea de Jos” University in Galați, 800216 Galați, Romania; sorinanicoleta.munteanu@yahoo.com (S.N.M.); nechitaaurel@yahoo.com (A.N.); dana_tutunaru_cmgl@yahoo.com (D.T.); nechitaluiza2012@yahoo.com (L.C.N.); aurelia.romila@yahoo.com (A.R.); 2 St. Apostle Andrei Clinical Emergency County Hospital, 800578 Galați, Romania; 3 St. Ioan Emergency Clinical Hospital for Children, 800487 Galați, Romania

**Keywords:** acute heart failure, carbohydrate antigen 125, CA 125, N-terminal pro b-type natriuretic peptide, NT-pro BNP, congestive heart failure

## Abstract

Background: Heart failure is a global major healthcare problem with millions of hospitalizations annually and with a very high mortality. There is an increased interest in finding new and reliable biomarkers for the diagnostic, prognostic and therapeutic guidance of patients hospitalized for acute heart failure; Our review aims to summarize in an easy-to-follow flow recent relevant research evaluating the possible use and the clinical value of measuring CA 125 serum levels in acute HF. Methods: A thorough search in the main international databases identified a relevant pool of 170 articles, providing recently published data for this narrative review that used PRISMA guidelines. Results: There are data to sustain the role of carbohydrate antigen 125 (CA 125), a worldwide used marker of ovarian cancer, in patients with heart failure. Several studies have shown links between CA 125 levels and congestion seen in acute heart failure, high mortality and readmission rates at 6 months follow-up after discharge from acute heart failure and also a role of CA 125 in the guidance of heart failure therapy. There are also clinical trials that showed that several particularities of CA 125 make it even better than N-terminal pro b-type natriuretic peptide (NT-pro BNP)—a classical and more utilized marker of heart failure) in several scenarios of acute heart failure. Conclusions: Although the mechanism behind the upregulation of serum CA 125 in patients with congestive HF has not been confirmed nor fully understood.

## 1. Introduction

Heart failure is still a current major healthcare concern worldwide, especially in the well-developed countries, such as the US and European countries, where heart failure is the third cause of cardiovascular mortality, with about 1 million hospitalizations annually [1,2].

Heart failure (HF) is a clinical syndrome characterized by a constellation of symptoms (dyspnea, orthopnea, lower limb swelling) and signs (elevated jugular venous pressure, pulmonary congestion) often caused by a structural and/or functional cardiac abnormality resulting in reduced cardiac output and/or elevated intracardiac pressures.

Acute heart failure (AHF) is broadly defined as a rapid onset of new or worsening signs and symptoms of heart failure. It is a potentially life-threatening condition, requiring hospitalization, and emergency treatment is aimed predominantly at managing fluid overload and hemodynamic compromise. AHF is a condition with an adverse prognosis, characterized by high mortality and rehospitalization rates and represents a significant financial burden to health systems.

Several circulating biomarkers have been studied for the use in appreciating the prognostic risk and the response to therapy in heart failure patients. Among these, the natriuretic peptides are the most used and studied biomarkers of heart failure [2,3,4].

Cancer Antigen 125 (CA 125) has recently emerged as a potential prognostic indicator and biomarker for guiding decongestive therapy in heart failure patients [4,5,6].

Carbohydrate antigen 125 (CA 125) is a high molecular weight transmembrane glycoprotein belonging to the mucin family, also known as MUC1644. It was first detected in ovarian cancer cells, but several studies showed that it is normally expressed on different cell surfaces present in various organs (lung, prostate, pleura, pericardium, peritoneum), with the role of hydrating and lubricating epithelial surfaces, thus protecting them from mechanical stress [7,8,9,10].

Clinically, it has been used as a marker of ovarian cancer, in monitoring, risk stratification and prognostication. CA 125 levels also rise in other malignancies such as lung cancer, mediastinal teratoma and non-Hodgkin lymphoma [11,12]. Although CA 125 is a well-known marker of ovarian cancer, its serum levels are also upregulated in multiple nonmalignant pathological states but also in physiologic conditions: pregnancy, menstruation, liver cirrhosis, pelvic inflammatory disease, peritoneal trauma, ascites, lung cancer and congestion due to heart failure [13,14]. The exact mechanism is not known but it is probably related to several mechanisms.

One proposed pathophysiological mechanism is the increased mechanical stress produced by excessive fluid accumulation. For example, CA 125 levels are positively correlated with pleural effusion volume in patients with chronic obstructive pulmonary disease and also with serosal fluid accumulation in patients with either benign or malignant diseases such as lung cancer or liver cirrhosis [15].

Inflammation seems to be another factor involved in the elevation of CA 125 levels in heart failure. We know that heart failure generates a systemic inflammatory state and that several proinflammatory cytokines (IL-6, IL-10, TNF alpha), are released in the blood circulation, and those cytokines can enhance the secretion of CA 125 [16]. Venous congestion produces changes in expression patterns of the endothelium and perivascular tissue, leading to upregulation of pro-oxidant, proinflammatory and vasoconstrictive factors. Bulska-Bedkowska et al. reported that CA 125 levels positively correlated with high-sensitivity C-reactive protein and IL-6 [8,17].

There are also data to suggest that CA 125 is implicated in the process of cardiac remodeling by modifying the intracellular matrix [18,19,20,21,22]. In patients hospitalized for acute heart failure, there was a positive correlation between galectin-3 and signs of inflammation only in patients with elevated CA 125 levels [18,22,23,24].

Duman et al. and Kouris et al. reported, in small studies, a weak association between CA 125 levels and pulmonary artery pressure, with no correlation to left side cardiac dysfunction. They also showed that CA 125 was associated with left atrial volume as an index of diastolic dysfunction [25,26].

Larger scale studies began to show the link between CA 125 and heart failure. D’ Aloia et al. [27] demonstrated a correlation between CA 125 levels and right heart dysfunction and left ventricular diastolic dysfunction. D’Aloia’s study had a larger total patient population and a wider range of heart failure severity, and thus more conclusive results. They also showed that lower levels of CA 125 at follow-up correlated with clinical improvement. In another large-scale study, Vizzardi et al. [28] reported that systolic and diastolic indices and cardiac diameter correlated with CA 125 levels.

More recent and also larger-scale studies performed by Nunez et al. demonstrated the predictive value of CA 125 for all-cause mortality at 6 months after acute heart failure discharge [29,30]. There is also a moderate correlation between CA 125 and natriuretic peptides, and the combination of the two seems to improve risk stratification of heart failure.

Our review aims to summarize in an easy-to-follow flow recent relevant research evaluating the possible use and the clinical value of measuring CA 125 serum levels in acute HF.

## 2. Methodology

**Figure 1:**
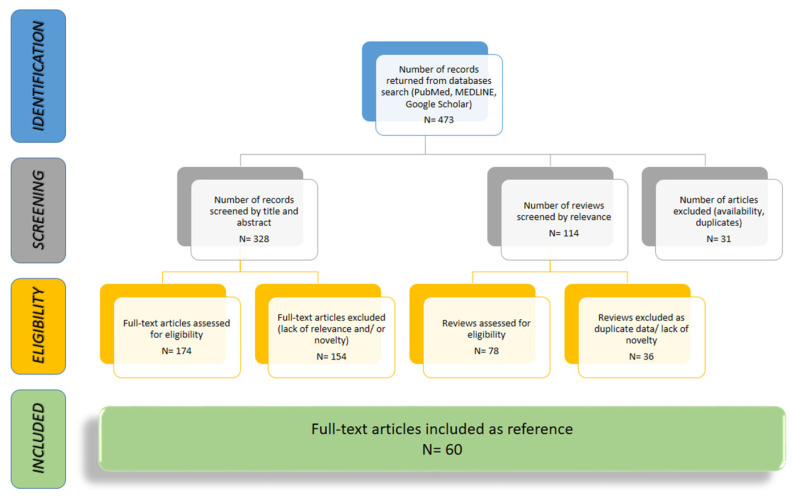
Flowchart of article selection after identification within databases.

Our literature search strategy was based on a thorough analysis aiming to identify articles on the selected topic, using the relevant key words, within broadly used databases and aggregators (PubMed, MEDLINE, Google Scholar). We performed this narrative review based on a relevant pool of over 200 articles selected after screening reviews, meta-analyses and randomized or observational clinical trials results published in the last 20 years (Figure 1).

The population included in these studies had a mean age of 70 years old and were diagnosed with heart failure according to the current guidelines of medical practice. Serum CA 125 levels were correlated with several clinical and imagistic parameters of heart failure and also with the short- and medium-term follow-up of patients after discharge.

This review is conducted following PRISMA guidelines. Although the search criteria started from a 20-year time span, we focused our work on presenting the latest data in order to maximize the clinical impact of the data.

## 3. Results

### 3.1. CA 125 and Congestion in Heart Failure

Congestion in heart failure is linked to poor outcome, as such we recognize the importance of early detection of congestion. However, the quantification of congestion can be quite challenging, especially in different settings such as in early phases of acute heart failure or close to discharge for hospitalization. Fluid overload and retention are the most common reasons for heart failure hospitalizations, and decongestion represents an important therapeutic target in these patients. However, complete decongestion in heart failure patients can be challenging, and so the residual congestion may be underappreciated leading to an increased risk of early rehospitalization and increased mortality [11,31,32,33,34,35].

CA 125 provides additional information regarding signs and symptoms of congestion, peripheral edema and serosal effusion in patients with heart failure. For example, in studies carried out by Falcao et al., in patients with heart failure complicating ST-elevation myocardial infarction, circulating CA 125 levels correlated with pulmonary congestion and had a similar prognostic power for mortality as N terminal pro B type natriuretic peptide (NT-pro BNP) [36,37,38]. Minana et al. showed that in cases of systemic congestion and right ventricular dysfunction CA 125 can outperform NT-pro BNP in the prediction of mortality [32,39].

Fluid retention and congestion are the primary reasons for hospitalization of heart failure patients. Therefore, congestion is an important therapeutic target and its evaluation is very important as it is often difficult.

There are two types of congestion that need to be evaluated in heart failure patients: intravascular congestion and tissue congestion. Underappreciation of congestion at discharge increases the risk of early rehospitalization and death in heart failure patients [40].

As such correct evaluation of residual congestion before discharge is very important, there is an increasing interest in the establishment of reliable and cost-effective biomarkers of fluid overload in heart failure [40,41].

CA 125 was studied as a potential biomarker of fluid overload in heart failure.

Studies conducted by Soler [42] and Minana [32] suggested that CA 125 may outperform NT-pro BNP in predicting mortality in cases of systemic congestion and right ventricular dysfunction. Minana et al. conducted a study of 2949 patients hospitalized for acute heart failure, in which they tried to determine the main factors associated with CA 125 and NT-pro BNP [32]. The median value for NT-pro BNP was 4840 pg/mL and for CA 125 was 58 U/mL. The main factors associated with NT-pro BNP levels were glomerular filtration rate, left ventricle ejection fraction and age, as opposed to CA 125 levels which were associated with presence of pleural effusion, tricuspid regurgitation severity and peripheral edema. In conclusion, CA1 25 was a more useful marker of right heart failure, being less influenced by age and renal function than NT-pro BNP [32].

In a prospective observational study including 191 patients admitted for acute heart failure, conducted by Pau Llacer et al. [43], CA 125 levels were associated more significantly than NT-pro BNP with the state of congestion. CA 125 was positively associated with signs of congestion such as: peripheral edema, pleural effusion and elevated inferior vena cava diameter. CA 125 was the most important predictor of inferior vena cava dilatation.

In another study, Gonzalo Nunez-Marin et al. aimed to determine if CA 125 and NT-pro BNP were associated with patterns of congestion, as measured by intrarenal venous flow Doppler ultrasound, in patients hospitalized with acute heart failure. A total of 70 patients with the mean age of 73 years were enrolled and renal Doppler ultrasound was assessed during the first 24 h of admission. CA 125, with a cut off value of 63.5 U/mL, showed a positive association with congestive renal ultrasound patterns and not NT-pro BNP [44].

In a meta-analysis performed by Li et al., including sixteen studies, with a total of 8401 patients with acute heart failure, high CA 125 levels were associated with acute heart failure symptoms and with more severe fluid overload [41].

Two studies have shown the relation between CA 125 and echocardiographic parameters of congestion and heart failure. D’Aloia et al. observed that CA 125 levels correlated with pulmonary artery pressure, right atrial pressure and deceleration time as measured by Doppler echocardiography [27]. Yilmaz et al. showed that CA 125 levels were negatively correlated with left ventricle ejection fraction and positively correlated with pulmonary artery pressure as measured by Doppler echocardiography [45].

### 3.2. CA 125 and Risk Stratification in Heart Failure

Apart from its role in evaluating congestion, CA 125 also seems to correlate with prognosis and mortality in heart failure patients.

In a retrospective trial including 2961 patients discharged after acute heart failure hospitalisation, with mean age of 74 years, Soler and Minana compared NT-pro BNP and CA 125 as predictors of poor outcome [42]. Both biomarkers were analysed in relation to the severity of tricuspid regurgitation (which is a factor of poor prognosis in heart failure). NT-pro BNP was found to be linearly linked to mortality in non severe tricuspid regurgitation, but not in severe tricuspid regurgitation. High CA 125 levels were associated with increased mortality especially in patients with more severe tricuspid regurgitation. In conclusion CA 125 may outperform NT-pro BNP in predicting mortality in heart failure patients with more involvement of the right ventricle and with more severe tricuspid regurgitation [42,46,47].

In the CHANCE-HF trial, conducted by Nunez et al., CA 125 emerged as a marker of prognosis in heart failure patients, by reducing the risk of acute heart failure readmissions when used to guide therapy after acute heart failure discharge [34].

Hung et al. conducted a trial including 158 female patients with acute heart failure and preserved ejection fraction. During follow-up, those with CA 125 levels > 17.29 U/mL had a greater incidence of heart failure rehospitalisation, and also CA 125 levels correlated with maximum left atrial volume [48].

A study performed by Nunez et al. sought to determine the CA 125 cut-point for identifying patients at low risk of 1 month death and composite death/heart failure readmissions. In patients with acute heart failure a serum level of CA 125 < 23 U/mL was corellated with low risk of adverse events after discharge. This cutoff value remained significant up to 6 months after discharge [34].

**Figure 2:**
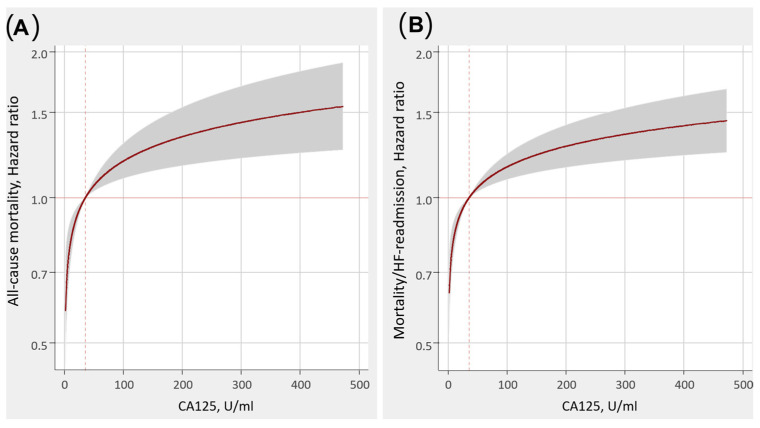
Correlation between CA 125 levels and mortality/heart failure readmissions (1-year multivariable analysis): (**A**)—All-cause mortality; (**B**)—Mortality/heart failure (HF) readmission (data from the BIOSTAT-CHF trial, 2020) [30].

In a subanalysis of BIOSTAT—CHF Study [30], there was an evaluation of the association between CA 125 and the risk of one-year clinical outcomes in patients with heart failure. Higher levels of CA 125 were associated with an increased risk of death and composite of death/heart failure readmissions (Figure 2).

As shown by Yoon et al., patients hospitalized for acute heart failure, with high levels of CA 125 and low levels of NT-pro BNP, had worse mid-term prognosis than patients with low levels of both CA 125 and NT-pro BNP and patients with high levels of both CA 125 and NT-pro BNP had the worse prognosis [49].

The most relevant publications presenting CA 125 involvement in heart failure risk stratification are presented in Table 1.

### 3.3. CA 125 Use in Guiding Heart Failure Therapy

One of the cornerstones of heart failure management is decongestion therapy. The treatment of fluid overload in heart failure is based on diuretics, although no trials have shown a mortality benefit from diuretic therapy in heart failure patients. Also, there is no clear strategy of diuretic therapy titration in these patients. The main interest is to escalate treatment intensity in patients with more volume overload and also to reduce diuretic doses in patients who would not benefit from more intense diuretic therapy, thus reducing adverse reactions.

Because of the link between CA 125 and the levels of fluid overload (congestion) in heart failure, CA 125 has emerged as a potential marker of guiding treatment in acute heart failure.

As shown by Nunez et al. in a trial of 946 patients with acute heart failure, where NT-pro BNP and CA 125 were measured repeatedly at each doctor-patient visit, the levels of CA 125 decreased in the subset of patients with lower risk, and in patients in whom the CA 125 levels remained high, the mortality risk was also high. The association between CA 125 and mortality risk in heart failure patients would argue for a role for CA 125 in tailoring decongestion therapy in these patients [40].

In the CHANCE-HF trial, the authors compared CA-125-guided therapy versus standard of care therapy in terms of composite of one year death/heart failure readmissions, in patients discharged after acute heart failure hospitalization. CA-125-guided therapy resulted in a reduction in the composite endpoint at one year follow-up, mainly by a significant reduction in acute heart failure readmissions, but had no effect on mortality [50].

Nunez et al. proposed an algorithm for the use of CA 125 in heart failure therapy guidance. Using data from the CHANCE-HF trial [50], a cutoff value for CA 125 was proposed at 35 U/mL. A total of 380 patients, discharged after acute heart failure hospitalization, with a high level of CA 125 > 35 U/mL, were assigned to CA-125-guided therapy versus standard of care therapy. In patients enrolled in CA-125-guided therapy the diuretic treatment was intensified when the CA 125 levels remained high or increased. Also, when CA 125 levels decreased below 35 U/mL, the diuretics were down-titrated. Those patients allocated to CA-125-guided therapy had 50% more diuretic dose modification that patients with standard of care treatment. The CA-125-guided therapy was superior to standard of care therapy in terms of reducing the composite of mortality/ acute heart failure readmissions at one year. Patients who had CA 125 levels above 35 U/mL at the first outpatient visit had a threefold increased risk of six months heart failure readmission [50,54,55].

In another study by Nunez et al. [29], 160 patients with mean age of 78 years, acute heart failure and renal dysfunction, were randomized into two groups: one with loop diuretic guided therapy using CA 125 levels and the second group with standard care therapy. After 72 h from admission, patients with CA-125-guided therapy, with CA 125 levels over 35 U/mL, received the highest doses of loop diuretics and had the highest volume depletion, with significant improvement of glomerular filtration rate as opposed to patients with the usual loop diuretic therapy. CA-125-guided diuretic therapy improved glomerular filtration rate at 72 h in patients admitted for acute heart failure and renal dysfunction [29].

## 4. Discussion

There are sufficient data to suggest that CA 125 has a potential role in the clinical workup of patients with acute heart failure, as a prognostic tool, a biomarker of congestion and a guide for decongestion therapy.

It appears that CA 125 is also involved in the processes of fluid and cell transport, inflammation, tissue repair and tumor dissemination. CA-125-associated N-glycans are involved in modulating immune responses; for example, CA 125 can suppress natural killer activity by the interaction with several proteins.

The exact mechanisms of CA-125-increased secretion in heart failure is not completely known. Nagele et al. firstly reported the findings of several relevant tumor markers, especially CA 125, in heart failure patients before and after heart transplantation [19,21].

As there is no other more appropriate biomarker that correlates with the congestive status of patients with acute heart failure, the role and benefits of CA 125 in acute heart failure patients are supported by some strong reasons. The first one would be that CA 125 has additional prognostic information beyond the classical biomarkers of heart failure (BNP, NT-pro BNP); the addition of CA 125 to NT-pro BNP may be a superior tool of risk estimation than NT-pro BNP alone. Another piece of evidence is the correlation of CA 125 serum levels with echocardiographic parameters of heart failure and with the right heart side involvement. It is proven that CA 125 has a longer half-life that NT-pro BNP, which makes it more stable and more reliable as a prognostic marker and most studies found it is not significantly impacted by factors such as age and renal dysfunction, as opposed to NT-pro BNP.

**Figure 3:**
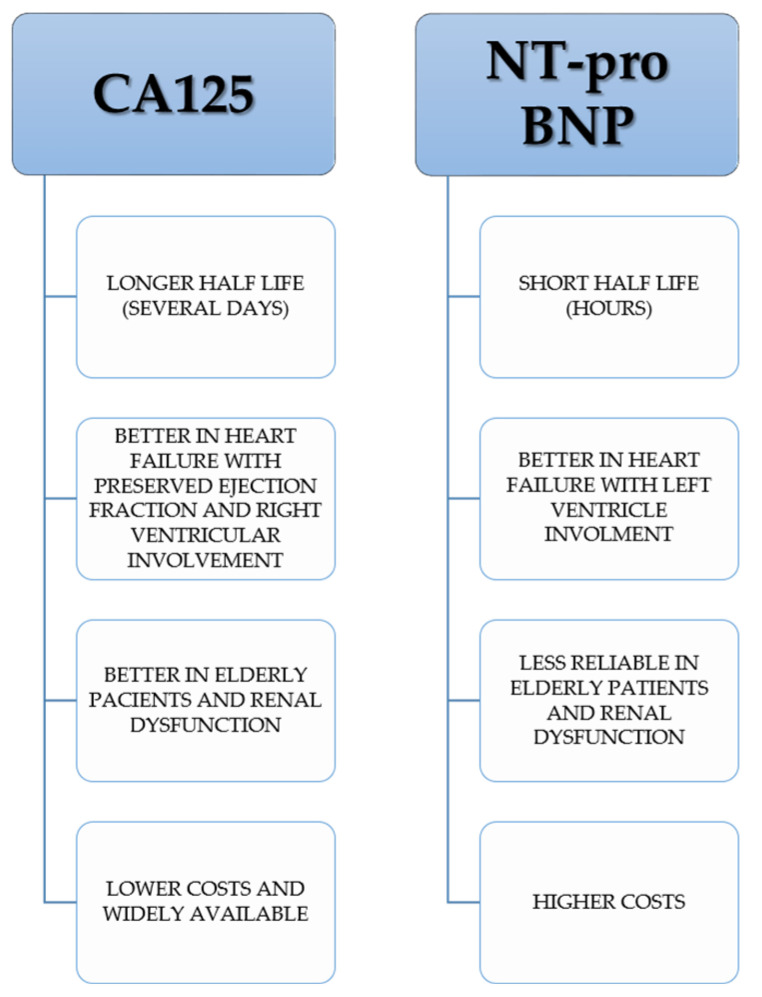
Main differences between CA 125 and NT-pro BNP use in heart failure.

CA 125 can have some advantages over NT-pro BNP (a biomarker of heart failure used on a larger scale) in circumstances such as heart failure with predominant involvement of right ventricle (especially heart failure with preserved ejection fraction), renal dysfunction and elderly patients [29,40,56]. Other advantages of CA 125 over NT-pro BNP can be of a logistical nature, e.g., CA 125 is widely available as it has already been used as a cancer marker for several decades and the cost of assessment is lower than for natriuretic peptides (Figure 3).

Cancer Antigen 125 (CA 125) should be measured at admission of patients with acute decompensated heart failure. As CA 125 is not a cardiac-specific biomarker, and its upregulation may occur in relation to other diseases, in the absence of a heart failure diagnosis, CA 125 levels should be interpreted accordingly.

Production of CA 125 in patients with heart failure is supposed to happen by increased mechanical stress in mesothelial cells, in response to hemodynamic and inflammation stimuli. Fluid overload and consecutive high venous pressure in heart failure may increase pressure in the mesothelium, which could induce the release of several inflammatory markers (IL-6, IL-10, tumor necrosis factors). This inflammation process along with the increased mechanical stress can stimulate mesothelial cells to secrete CA 125. Congestion in heart failure can involve pulmonary congestion, pleural effusion or ascites, which are interrelated with systemic inflammationin a vicious cycle [32,33].

The capacity of the mesothelium to secrete CA 125 was investigated and demonstrated by Zeillemaker et al., using a mesothelial cell monolayer model in vitro and utilizing inflammatory cytokines such as interleukin 1, tumor necrosis factor alpha and lipopolysaccharides as stimuli [16]. Peak secretion of CA 125 was observed in 6 h, and the most effective stimuli was IL-1.

As an additional mechanism, the translocation of bacteria and endotoxin formation during acute heart failure with bowel congestion leading to gastrointestinal functional impairment may play a role in the secretion of CA 125 [34]. Seo et al. observed a link between pericardial stimuli and elevation of CA 125 in patients with heart failure [35].

There is also a difference between patients with acute and stable heart failure. Up to two-thirds of patients with acute decompensated heart failure have elevated CA 125 levels (above 35 U/mL) but only a few patients with stable heart failure have CA 125 levels above this cutoff value. As such it is important to correlate CA 125 levels with signs and symptoms, echocardiographic parameters and other biomarkers such as natriuretic peptides [1,13,19,20].

A cutoff value of 35 U/mL was proposed for CA 125 levels that can identify low risk of adverse events following acute heart failure admission. High CA 125 levels (above 35 U/mL) can identify high risk patients and may guide a more intensive decongestion therapy with higher doses of loop diuretics.

It appears that during the first months after acute heart failure hospitalization, CA 125 kinetics are correlated with the patients’ clinical outcome. Because CA 125 has a long half-life (several days as opposed to NT-pro BNP which has a half-life of hours), it is reasonable to measure CA 125 levels at admission and at least seven days after the initial measurement, for obtaining information about the response to therapy.

Although there are many studies that show the role of CA 125 in acute heart failure patients, there are fewer data about CA 125 in chronic ambulatory heart failure. One study, performed by Kieran F. Docherty et al. and published in The Journal of the American College of Cardiology in 2023, addressed this issue [53]. They examined the association between baseline CA 125 levels and outcome in patients from the DAPA-HF trial (Dapagliflozin and Prevention of Adverse Outcomes in Heart Failure) and also the relationship with the effect of dapagliflozin. The DAPA-HF was a prospective, randomized trial that examined the efficacy and safety of dapagliflozin 10 mg per day compared with placebo, in patients with heart failure with reduced ejection fraction. The patients included in this trial had stable, non-decompensated heart failure with reduced ejection fraction (LVEF < 40%). CA 125 was measured at baseline and after 12 months after randomization. CA 125 was analyzed according to the upper normal limit of 35 U/mL. Patients with higher concentrations of CA 125 were older, had worse NYHA functional class, higher heart rate and also higher NT-pro BNP and hs troponin T levels. They also exhibited lower left ventricular ejection fraction and more renal impairment. Higher CA 125 levels also correlated with more peripheral congestion. A history of atrial fibrillation was associated with more elevated CA 125 levels. Using the cutoff value of 35 U/mL, patients with elevated CA 125 concentrations had higher risks of primary and secondary morbidity/mortality outcomes. The risk of death caused by worsening heart failure was also higher in patients with CA 125 levels above 35 U/mL. Renal function also declined at a greater rate over time in patients with elevated CA 125. CA 125 levels in the DAPA-HF trial were lower than in patients with acute heart failure, with only 12% of patients having levels above the cutoff value of 35 U/mL [55,56,57,58]. Elevated levels of CA 125 at baseline and also an increase in CA 125 levels from baseline to 12 months were independent predictors of the risk of worsening heart failure and mortality. The effects of dapagliflozin were not influenced by the levels of CA 125 [52,53,57,58,59,60].

Further evidence and more in-depth data analysis coming from prospective studies is needed before we can see CA 125 evaluation included in the guidelines for the management of HF. We noted that most evidence we found in the literature comes from trials including selected populations, or with a relatively small sample size. Registry data are important but they do not provide serial measurements nor a more complete overview of dynamics and comparison with other biomarkers (like NT-pro BNP). Because a biomarker should have a sufficient specificity—which is not the case for CA 125, elevated in several non-HF-related conditions—and because we have limited knowledge on the pathophysiologic mechanisms of CA 125 involvement in HF, broad recommendations of its use in the clinical practice may not come very soon. In estimating cardiac congestion and short-term prognosis, the measurement of CA 125 concentrations should be complemented by the clinical information obtained from physical exam, natriuretic peptide levels and echocardiographic findings. Especially in older patients with impaired renal function and predominant involvement of the right side of the heart, an elevated CA 125 may guide the clinician to use intensive decongestion strategies (i.e., a more aggressive diuretic approach), so the clinical value of monitoring CA 125 may be more important for particular patients’ subgroups—but more research is needed.

## 5. Conclusions

CA 125 is a promising biomarker of congestion in the setting of acute heart failure and there is evidence to support its role in risk stratification, monitoring and guiding therapy in acute heart failure. More studies are necessary for the implementation of CA 125 in the clinical practice of heart failure management, to establish reference ranges for heart failure and appropriate algorithms for diagnosis and treatment monitoring.

While it is commonly available, for a better clinical effectiveness in screening and early detection CA 125 serum levels should be used by physicians in combination with both clinical manifestations as well as with other biomarkers or ultrasound and other multimodal methods.

## Figures and Tables

**Table 1:** Relevant publications on CA 125 involvement in the stratification of heart failure risk.

Author	Year	Number of Patients	Results
D’Aloia et al. [27]	2003	286	CA 125 > 35 U/mL—increased risk of death/repeated hospitalization at 6 months follow-up
Hung et al. [48]	2012	158	In acute heart failure patients with preserved ejection fraction CA 125 levels > 17.29 U/mL correlated with increased readmissions
Nunez et al. CHANCE-HF TRIAL [50]	2016	380	CA-125-guided therapy superior to standard of care therapy in terms of reducing 1 year risk of death/readmissions
Li et al. [41]	2018	8401	Increased CA 125 levels linked to higher incidence of death/readmission for acute heart failure
Nunez et al. BIOSTAT—CHF TRIAL [30]	2020	2516	CA 125 high levels correlated with 1 year risk of all cause mortality
Soler et al. [42]	2020	2961	Higher CA 125 levels in patients with high mortality risk
Lorenzo et al. [51]	2021	1387	Higher levels of CA-125 in patients with AHF may identify the need for a prolonged hospitalization
Minana et al. [52]	2022	4812	CA 125 positively associated with heart failure readmission risk
Docherty et al. [53]	2023	3123	Elevated CA 125 levels were an independent predictor of the risk of worsening heart failure or cardiovascular death

## References

[1] Hollenberg S.M., Warner Stevenson L., Ahmad T., Amin V.J., Bozkurt B., Butler J., Davis L.L., Drazner M.H., Kirkpatrick J.N., Peterson P.N. (2019). 2019 ACC Expert Consensus Decision Pathway on Risk Assessment, Management, and Clinical Trajectory of Patients Hospitalized with Heart Failure: A Report of the American College of Cardiology Solution Set Oversight Committee. J. Am. Coll. Cardiol..

[2] Rogers J.K., Pocock S.J., McMurray J.J., Granger C.B., Michelson E.L., Östergren J., Pfeffer M.A., Solomon S.D., Swedberg K., Yusuf S. (2014). Analysing recurrent hospitalizations in heart failure: A review of statistical methodology, with application to CHARM-Preserved. Eur. J. Heart Fail..

[3] Ma W., Zhang P., Hu H. (2023). Serum Levels of Hcy, sST2 and CA-125 in CHF Patients and Their Correlation with Cardiac Function Classification. Heart Surg. Forum..

[4] Pandhi P., Ter Maaten J.M., Anker S.D., Ng L.L., Metra M., Samani N.J., Lang C.C., Dickstein K., de Boer R.A., van Veldhuisen D.J. (2022). Pathophysiologic Processes and Novel Biomarkers Associated with Congestion in Heart Failure. JACC Heart Fail..

[5] Santas E., Palau P., Bayés-Ge A., Núñez J. (2020). The emerging role of carbohydrate antigen 125 in heart failure. Biomark. Med..

[6] Feng R., Zhang Z., Fan Q. (2023). Carbohydrate antigen 125 in congestive heart failure: Ready for clinical application?. Front. Oncol..

[7] Huang F., Chen J., Liu Y., Zhang K., Wang J., Huang H. (2012). New mechanism of elevated CA125 in heart failure: The mechanical stress and inflammatory stimuli initiate CA125 synthesis. Med. Hypotheses.

[8] Lei Y., Zang R., Lu Z., Zhang G., Huang J., Liu C., Wang Z., Mao S., Che Y., Wang X. (2020). ERO1L promotes IL6/sIL6R signaling and regulates MUC16 expression to promote CA125 secretion and the metastasis of lung cancer cells. Cell Death Dis..

[9] Scholler N., Urban N. (2007). CA125 in ovarian cancer. Biomark. Med..

[10] Lloyd K.O., Yin B.W. (2001). Synthesis and secretion of the ovarian cancer antigen CA 125 by the human cancer cell line NIH:OVCAR-3. Tumour Biol..

[11] Bottoni P., Scatena R. (2015). The Role of CA 125 as Tumor Marker: Biochemical and Clinical Aspects. Adv. Exp. Med. Biol..

[12] Miralles C., Orea M., España P., Provencio M., Sánchez A., Cantos B., Cubedo R., Carcereny E., Bonilla F., Gea T. (2003). Cancer antigen 125 associated with multiple benign and malignant pathologies. Ann. Surg. Oncol..

[13] Núñez J., Miñana G., Núñez E., Chorro F.J., Bodí V., Sanchis J. (2014). Clinical utility of antigen carbohydrate 125 in heart failure. Heart Fail. Rev..

[14] Llàcer P., Bayés-Genís A., Núñez J. (2019). Carbohydrate antigen 125 in heart failure. New era in the monitoring and control of treatment. Med. Clin..

[15] Topalak O., Saygili U., Soyturk M., Karaca N., Batur Y., Uslu T., Erten O. (2002). Serum, pleural effusion, and ascites CA-125 levels in ovarian cancer and nonovarian benign and malignant diseases: A comparative study. Gynecol. Oncol..

[16] Zeillemaker A.M., Verbrugh H.A., Hoynck van Papendrecht A.A., Leguit P. (1994). CA 125 secretion by peritoneal mesothelial cells. J. Clin. Pathol..

[17] Bulska-Będkowska W., Chełmecka E., Owczarek A.J., Mizia-Stec K., Witek A., Szybalska A., Grodzicki T., Olszanecka-Glinianowicz M., Chudek J. (2019). CA125 as a Marker of Heart Failure in the Older Women: Population-Based Analysis. J. Clin. Med..

[18] Núñez J., Rabinovich G.A., Sandino J., Mainar L., Palau P., Santas E., Villanueva M.P., Núñez E., Bodí V., Chorro F.J. (2015). Prognostic value of the interaction between galectin-3 and antigen carbohydrate 125 in acute heart failure. PLoS ONE.

[19] Nägele H., Bahlo M., Klapdor R., Schaeperkoetter D., Rödiger W. (1999). CA 125 and its relation to cardiac function. Am. Heart J..

[20] Núñez J., Núñez E., Consuegra L., Sanchis J., Bodí V., Martínez-Brotons A., Bertomeu-González V., Robles R., Bosch M.J., Fácila L. (2007). Carbohydrate antigen 125: An emerging prognostic risk factor in acute heart failure?. Heart.

[21] Núñez J., Miñana G., González M., Garcia-Ramón R., Sanchis J., Bodí V., Núñez E., Chorro F.J., Llàcer A., Miguel A. (2011). Antigen carbohydrate 125 in heart failure: Not just a surrogate for serosal effusions?. Int J Cardiol..

[22] Miñana G., Núñez J., Sanchis J., Bodí V., Núñez E., Llàcer A. (2010). CA125 and immunoinflammatory activity in acute heart failure. Int. J. Cardiol..

[23] Colombo P.C., Onat D., Harxhi A., Demmer R.T., Hayashi Y., Jelic S., LeJemtel T.H., Bucciarelli L., Kebschull M., Papapanou P. (2014). Peripheral venous congestion causes inflammation, neurohormonal, and endothelial cell activation. Eur. Heart J..

[24] Murphy S.P., Kakkar R., McCarthy C.P., Januzzi J.L. (2020). Inflammation in Heart Failure: JACC State-of-the-Art Review. J. Am. Coll. Cardiol..

[25] Duman D., Palit F., Simsek E., Bilgehan K. (2008). Serum carbohydrate antigen 125 levels in advanced heart failure: Relation to B-type natriuretic peptide and left atrial volume. Eur. J. Heart Fail..

[26] Kouris N.T., Zacharos I.D., Kontogianni D.D., Goranitou G.S., Sifaki M.D., Grassos H.E., Kalkandi E.M., Babalis D.K. (2005). The significance of CA125 levels in patients with chronic congestive heart failure. Correlation with clinical and echocardiographic parameters. Eur. J. Heart Fail..

[27] D’Aloia A., Faggiano P., Aurigemma G., Bontempi L., Ruggeri G., Metra M., Nodari S., Dei Cas L. (2003). Serum levels of carbohydrate antigen 125 in patients with chronic heart failure: Relation to clinical severity, hemodynamic and Doppler echocardiographic abnormalities, and short-term prognosis. J. Am. Coll. Cardiol..

[28] Vizzardi E., D’Aloia A., Curnis A., Dei Cas L. (2013). Carbohydrate antigen 125: A new biomarker in heart failure. Cardiol. Rev..

[29] Núñez J., Llàcer P., García-Blas S., Bonanad C., Ventura S., Núñez J.M., Sánchez R., Fácila L., de la Espriella R., Vaquer J.M. (2020). CA125-Guided Diuretic Treatment Versus Usual Care in Patients with Acute Heart Failure and Renal Dysfunction. Am. J. Med..

[30] Núñez J., Bayés-Genís A., Revuelta-López E., Ter Maaten J.M., Miñana G., Barallat J., Cserkóová A., Bodi V., Fernández-Cisnal A., Núñez E. (2020). Clinical Role of CA125 in Worsening Heart Failure: A BIOSTAT-CHF Study Subanalysis. JACC Heart Fail..

[31] Kumric M., Kurir T.T., Bozic J., Glavas D., Saric T., Marcelius B., D’Amario D., Borovac J.A. (2021). Carbohydrate Antigen 125: A Biomarker at the Crossroads of Congestion and Inflammation in Heart Failure. Card. Fail. Rev..

[32] Miñana G., de la Espriella R., Mollar A., Santas E., Núñez E., Valero E., Bodí V., Chorro F.J., Fernández-Cisnal A., Martí-Cervera J. (2020). Factors associated with plasma antigen carbohydrate 125 and amino-terminal pro-B-type natriuretic peptide concentrations in acute heart failure. Eur. Heart J. Acute Cardiovasc. Care..

[33] Núñez J., Sanchis J., Bodí V., Fonarow G.C., Núñez E., Bertomeu-González V., Miñana G., Consuegra L., Bosch M.J., Carratalá A. (2010). Improvement in risk stratification with the combination of the tumour marker antigen carbohydrate 125 and brain natriuretic peptide in patients with acute heart failure. Eur. Heart J..

[34] Núñez J., Bayés-Genís A., Revuelta-López E., Miñana G., Santas E., Ter Maaten J.M., de la Espriella R., Carratalá A., Lorenzo M., Palau P. (2022). Optimal carbohydrate antigen 125 cutpoint for identifying low-risk patients after admission for acute heart failure. Rev. Esp. Cardiol..

[35] Seo T., Ikeda Y., Onaka H., Hayashi T., Kawaguchi K., Kotake C., Toda T., Kobayashi K. (1993). Usefulness of serum CA125 measurement for monitoring pericardial effusion. Jpn. Circ. J..

[36] Falcão F.J.A., Oliveira F.R.A., Cantarelli F., Cantarelli R., Brito-Júnior P., Lemos H., Silva P., Camboim I., Freire M.C., Carvalho O. (2019). Carbohydrate antigen 125 predicts pulmonary congestion in patients with ST-segment elevation myocardial infarction. Braz. J. Med. Biol. Res..

[37] Falcão F., Oliveira F., Cantarelli F., Cantarelli R., Brito Júnior P., Lemos H., Silva P., Camboim I., Freire M.C., Carvalho O. (2020). Carbohydrate antigen 125 for mortality risk prediction following acute myocardial infarction. Sci. Rep..

[38] Bobeica C., Niculet E., Tatu A.L., Craescu M., Vata D., Statescu L., Iancu A.V., Musat C.L., Draganescu M.L., Onisor C. (2022). Old and new therapeutic strategies in systemic sclerosis (Review). Exp. Ther. Med..

[39] Miñana Escrivá G., Núñez J., Sanchis J., Bodi V., Núñez E., Chorro F.J., Llàcer A. (2012). Mediciones seriadas de antígeno carbohidrato 125 tras un ingreso por insuficiencia cardiaca aguda y riesgo de reingreso precoz [Carbohydrate antigen 125 serial measurements after an admission for acute heart failure and risk of early readmission]. Med. Clin..

[40] Núñez J., Núñez E., Bayés-Genís A., Fonarow G.C., Miñana G., Bodí V., Pascual-Figal D., Santas E., Garcia-Blas S., Chorro F.J. (2017). Long-term serial kinetics of N-terminal pro B-type natriuretic peptide and carbohydrate antigen 125 for mortality risk prediction following acute heart failure. Eur. Heart J. Acute Cardiovasc. Care..

[41] Li K.H.C., Gong M., Li G., Baranchuk A., Liu T., Wong M.C.S., Jesuthasan A., Lai R.W.C., Lai J.C.L., Lee A.P.W. (2018). International Health Informatics Study (IHIS) Network. Cancer antigen-125 and outcomes in acute heart failure: A systematic review and meta-analysis. Heart Asia.

[42] Soler M., Miñana G., Santas E., Núñez E., de la Espriella R., Valero E., Bodí V., Chorro F.J., Fernández-Cisnal A., D’Ascoli G. (2020). CA125 outperforms NT-proBNP in acute heart failure with severe tricuspid regurgitation. Int. J. Cardiol..

[43] Llàcer P., Gallardo M.Á., Palau P., Moreno M.C., Castillo C., Fernández C., de la Espriella R., Mollar A., Santas E., Miñana G. (2021). Comparison between CA125 and NT-proBNP for evaluating congestion in acute heart failure. Med. Clin..

[44] Núñez-Marín G., de la Espriella R., Santas E., Lorenzo M., Miñana G., Núñez E., Bodí V., González M., Górriz J.L., Bonanad C. (2021). CA125 but not NT-proBNP predicts the presence of a congestive intrarenal venous flow in patients with acute heart failure. Eur. Heart J. Acute Cardiovasc. Care..

[45] Yilmaz M.B., Zorlu A., Tandogan I. (2011). Plasma CA-125 level is related to both sides of the heart: A retrospective analysis. Int. J. Cardiol..

[46] Oprea V.D., Marinescu M., Rișcă Popazu C., Sârbu F., Onose G., Romila A. (2022). Cardiovascular Comorbidities in Relation to the Functional Status and Vitamin D Levels in Elderly Patients with Dementia. Diagnostics..

[47] Menghoum N., Badii M.C., Deltombe M., Lejeune S., Roy C., Vancraeynest D., Pasquet A., Gerber B.L., Horman S., Gruson D. (2024). Carbohydrate antigen 125: A useful marker of congestion, fibrosis, and prognosis in heart failure with preserved ejection fraction. ESC Heart Fail..

[48] Hung C.L., Hung T.C., Lai Y.H., Lu C.S., Wu Y.J., Yeh H.I. (2013). Beyond malignancy: The role of carbohydrate antigen 125 in heart failure. Biomark. Res..

[49] Yoon J.Y., Yang D.H., Cho H.J., Kim N.K., Kim C.Y., Son J., Roh J.H., Jang S.Y., Bae M.H., Lee J.H. (2019). Serum levels of carbohydrate antigen 125 in combination with N-terminal pro-brain natriuretic peptide in patients with acute decompensated heart failure. Korean J. Intern. Med..

[50] Núñez J., Llàcer P., Bertomeu-González V., Bosch M.J., Merlos P., García-Blas S., Montagud V., Bodí V., Bertomeu-Martínez V., Pedrosa V. (2016). CHANCE-HF Investigators. Carbohydrate Antigen-125-Guided Therapy in Acute Heart Failure: CHANCE-HF: A Randomized Study. JACC Heart Fail..

[51] Lorenzo M., Palau P., Llàcer P., Domínguez E., Ventura B., Núñez G., Miñana G., Solsona J., Santas E., De La Espriella R. (2021). Clinical utility of antigen carbohydrate 125 for planning the optimal length of stay in acute heart failure. Eur. J. Intern. Med..

[52] Miñana G., de la Espriella R., Palau P., Llácer P., Núñez E., Santas E., Valero E., Lorenzo M., Núñez G., Bodí V. (2022). Carbohydrate antigen 125 and risk of heart failure readmissions in patients with heart failure and preserved ejection fraction. Sci. Rep..

[53] Docherty K.F., McDowell K., Welsh P., Osmanska J., Anand I., de Boer R.A., Køber L., Kosiborod M.N., Martinez F.A., O’Meara E. (2023). Association of Carbohydrate Antigen 125 on the Response to Dapagliflozin in Patients with Heart Failure. J. Am. Coll. Cardiol..

[54] Núñez J., Llàcer P., Núñez E., Ventura S., Bonanad C., Bodí V., Miñana G., Santas E., Mascarell B., Fonarow G.C. (2014). Antigen carbohydrate 125 and creatinine on admission for prediction of renal function response following loop diuretic administration in acute heart failure. Int. J. Cardiol..

[55] Núñez J., Núñez E., Miñana G., Bodí V., Fonarow G.C., Bertomeu-González V., Palau P., Merlos P., Ventura S., Chorro F.J. (2012). Differential mortality association of loop diuretic dosage according to blood urea nitrogen and carbohydrate antigen 125 following a hospitalization for acute heart failure. Eur. J. Heart Fail..

[56] Ordu S., Ozhan H., Alemdar R., Aydin M., Caglar O., Yuksel H., Kandis H. (2012). Carbohydrate antigen-125 and N-terminal pro-brain natriuretic peptide levels: Compared in heart-failure prognostication. Tex. Heart Inst. J..

[57] de la Espriella-Juan R., Núñez E., Sanchis J., Bayés-Genis A., Núñez J. (2018). Carbohydrate Antigen-125 in Heart Failure: An Overlooked Biomarker of Congestion. JACC Heart Fail..

[58] Núñez J., de la Espriella R., Miñana G., Santas E., Llácer P., Núñez E., Palau P., Bodí V., Chorro F.J., Sanchis J. (2021). Antigen carbohydrate 125 as a biomarker in heart failure: A narrative review. Eur. J. Heart Fail..

[59] Sikaris K.A. (2011). CA125—A test with a change of heart. Heart Lung Circ..

[60] Xu K., Wu M., Huang M., Zhuo X., Weng Y., Chen X. (2022). Carbohydrate antigen 125 combined with N-terminal pro-B-type natriuretic peptide in the prediction of acute heart failure following ST-elevation myocardial infarction. Medicine.

